# Focal endocardial pulsed-field ablation for the treatment of inappropriate sinus tachycardia: A case report

**DOI:** 10.1016/j.hrcr.2025.05.022

**Published:** 2025-05-30

**Authors:** Mohamed A. Mostafa, Jonathan R. Brock, Edward H. Kincaid, Prashant D. Bhave, S. Patrick Whalen

**Affiliations:** 1 Section on Cardiovascular Medicine, Department of Internal Medicine, Wake Forest School of Medicine, Winston-Salem, North Carolina; 2 Department of Cardiothoracic Surgery, Wake Forest School of Medicine, Winston-Salem, North Carolina

**Keywords:** Dual-energy focal catheter, Endocardial, Inappropriate sinus tachycardia, Phrenic nerve, Pulsed-field ablation

**Key Teaching Points**

• Focal pulsed-field ablation (PFA) can be safely applied in close proximity to the phrenic nerve for sinus node modification in patients with inappropriate sinus tachycardia (IST), as demonstrated by the absence of phrenic nerve injury in this case. This highlights the potential of PFA as a tissue-specific ablation modality in anatomically sensitive areas.
• The long-term efficacy of this approach may be limited, as evidenced by the recurrence of symptoms within a few months in this patient. This underscores the ongoing challenges in achieving durable sinus node modification, particularly via an endocardial approach.
• Further research is warranted to optimize the long-term outcomes of PFA for IST, including exploring more aggressive ablation strategies, adjunctive techniques, and a better understanding of lesion characteristics and tissue response to PFA in the sinoatrial node region to improve the durability of treatment.

## Introduction

Inappropriate sinus tachycardia (IST) is a diagnosis of exclusion, characterized by debilitating symptoms such as a markedly elevated heart rate at rest or with minimal exertion, along with exercise intolerance. The condition is defined by a resting heart rate exceeding 100 beats per minute (bpm) and a mean ambulatory heart rate of >90 bpm without a clear secondary cause.^1^ Although the exact pathophysiology remains unclear, IST is believed to be multifactorial, involving abnormal neurohumoral modulation, antibody-mediated processes, and various receptor hyper- or hyposensitivity mechanisms.^2^ Primarily affecting women of childbearing age, IST remains challenging to manage owing to high symptomatic recurrence rates despite lifestyle modifications, medical therapies (eg, beta blockers, ivabradine), and ablation techniques.^3^, ^4^, ^5^

Catheter ablation is considered a primary treatment option for patients with ectopic atrial tachycardia and an adjunctive treatment in the management of patients with IST.^5^ However, conventional radiofrequency (RF) ablation and cryoablation in the area of interest are not tissue specific and carry a risk of extracardiac collateral damage, such as esophageal and phrenic nerve injuries.^6^ Pulsed-field ablation (PFA) is a nonthermal tissue-specific modality that is currently only approved for pulmonary vein isolation in the United States. Some case reports successfully used PFA outside the pulmonary veins.^7^ It has been associated with a lower incidence of phrenic nerve injury.^8^^,^^9^

Given the challenges with patients with IST, there is increasing interest in exploring innovative technologies and strategies to improve outcomes. In this report, we present our approach to a complex case of IST managed with contact force and 3-dimensional (3D) integrated focal PFA near the phrenic nerve, highlighting the acute procedural efficacy and safety of this innovative approach. Informed consent was obtained from the patient before the use of the investigational device, ensuring they were fully aware of the nature, potential risks, and benefits associated with the procedure.

## Case report

Our patient is a 24-year-old white woman with a history of IST who previously underwent a hybrid surgical epicardial and endocardial ablation of the high crista terminalis/superior vena cava for sinus node modification 14 months ago. The procedure was performed via a right thoracoscopic approach under general anesthesia with no isoproterenol testing used during the case. In addition, the patient had both typical atrioventricular nodal reentrant tachycardia and cavotricuspid isthmus–dependent right atrial flutter induced during the procedure and slow pathway modification (right inferior extension), and cavotricuspid isthmus ablation was performed using RF. After the procedure, the patient continued to endorse chest pain, exercise intolerance, and frequent palpitations with an average heart rate of >100 bpm on ambulatory cardiac monitors. Planned subsequent endocardial procedure was halted because areas of earliest atrial activation targeted for ablation were in close proximity to the right phrenic nerve, with phrenic nerve stimulation seen during high-output pacing (20 mA, 2 ms pulse width). Her treatment options were discussed, including pericardial manipulation of the phrenic nerve and focal endocardial ablation with PFA once available. She continued to have significant dyspnea on exertion, resting tachycardia, and chest pain despite optimal medical therapy on beta blocker and ivabradine.

Compassionate use of PFA using an investigational dual-energy (RF and PFA) platform (Dual Energy THERMOCOOL SMARTTOUCH SF Catheter and TRUPULSE Generator; Biosense Webster Inc, part of Johnson & Johnson MedTech, Irvine, CA) was requested and ultimately granted by the manufacturer. The procedure was done under general anesthesia.

**Figure 1:**
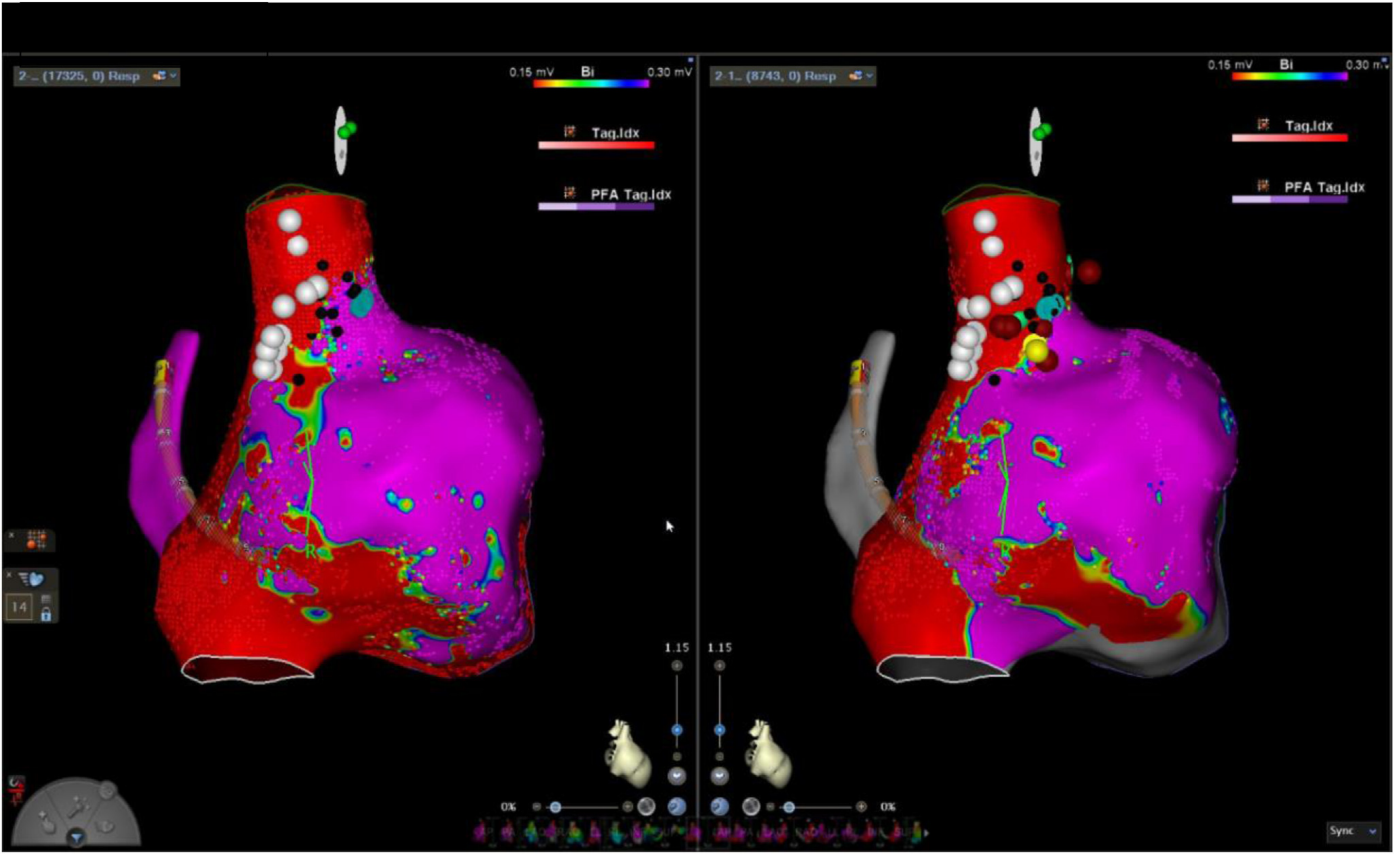
Preablation and postablation voltage maps with *white dots* signifying areas of phrenic nerve capture with high-output pacing and *black dots* representing sites in which the phrenic nerve was not captured during pacing.

The patient presented for endocardial activation mapping and ablation. The patient’s sinus rate at the start of the procedure ranged from 100 to 110 bpm. Earliest atrial activation was mapped at baseline and during incremental isoproterenol infusion at 1 μg/min and 2 μg/min. There was no significant change in either sinus rate or activation pattern between baseline and the 2 isoproterenol doses. Areas of phrenic nerve stimulation were also assessed with high-output pacing and superimposed on the electroanatomic map (Figure 1).

**Figure 2:**
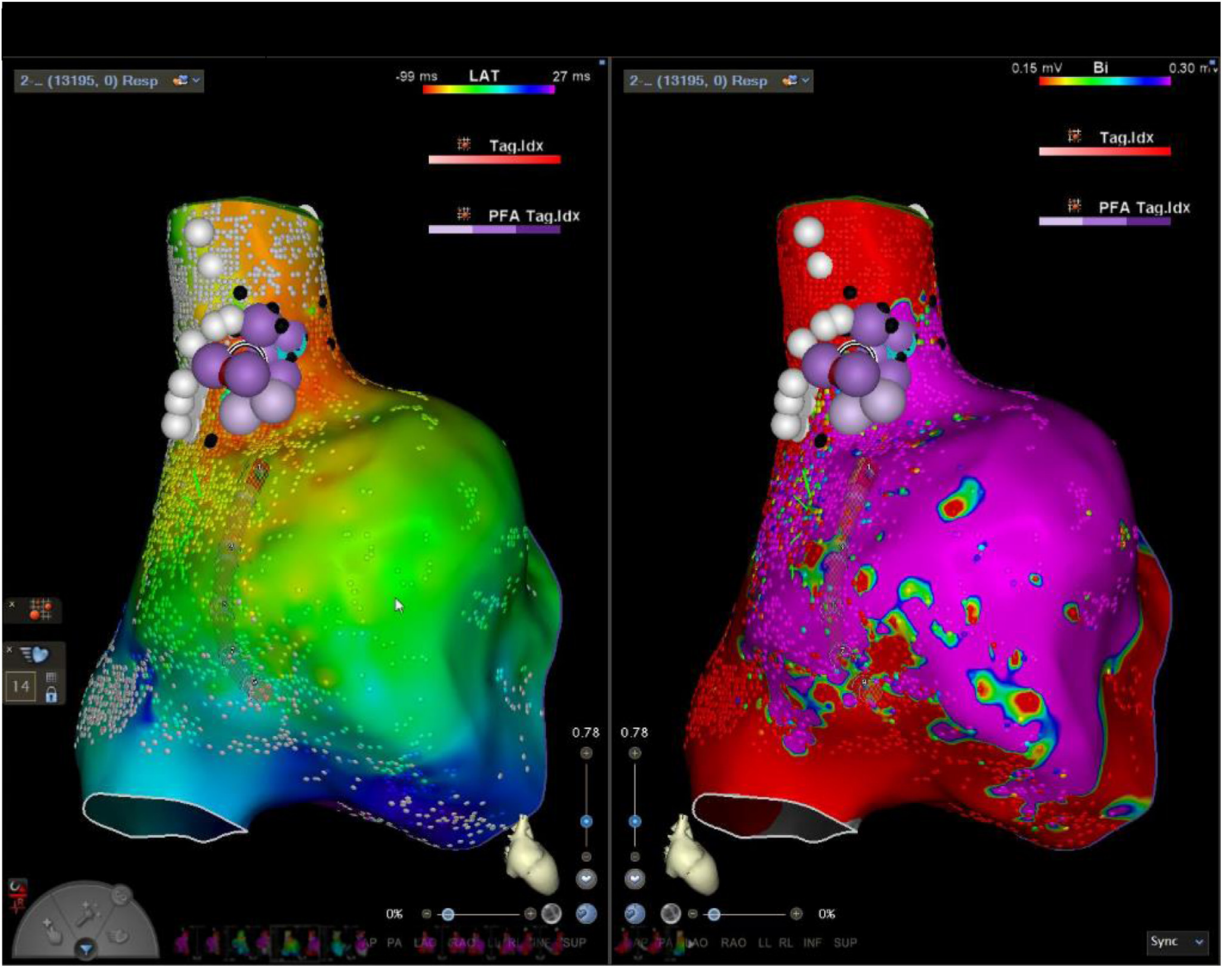
Activation map (left) and voltage map (right) with delivered applications of pulsed-field ablation (*purple dots*) and areas of phrenic nerve capture (*white dots*) on 2 mg of isoproterenol.

**Figure 3:**
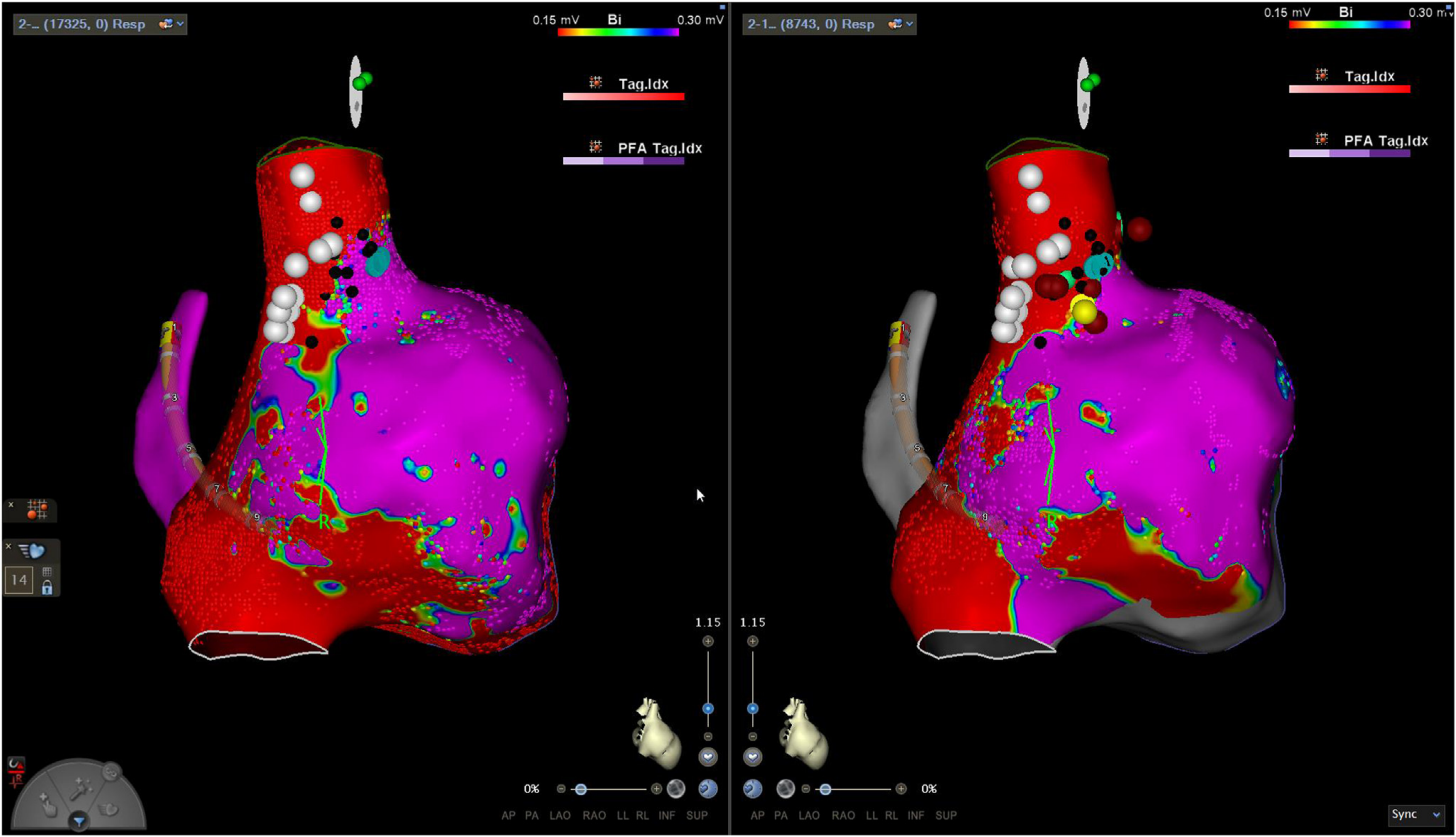
Preablation and postablation voltage map at baseline off isoproterenol.

Eight PFA applications were applied over the areas of earliest atrial activation in close proximity to the right phrenic nerve. Each application delivered repeated pulsed-field energy, with the number of pulses per application ranging from 12 to 24, with a maximum of 24 pulses per application, aligning with the safety goal of minimizing phrenic nerve injury while ensuring effective lesion formation with PFA. The average contact force during PFA delivery was 10 g, with a minimum of 5 g and a maximum of 19 g. Phrenic nerve capture was evaluated at both the beginning and end of the procedure, with bilateral diaphragmatic motion confirmed on fluoroscopy during spontaneous breathing. However, capture was not assessed after each PFA application, so transient effects on phrenic nerve function during intermediate stages of the procedure cannot be definitively determined. A visual interlesion ablation tag distance of 6–8 mm was consistently maintained. During PFA application, an immediate reduction in heart rate was noted multiple times with a junctional rhythm (Figures 2 and 3).

After the final applications, phrenic nerve capture was reassessed, and bilateral diaphragmatic movement with spontaneous breathing was again appreciated on fluoroscopy. After cessation of isoproterenol and conclusion of the case, the patient exhibited an accelerated junctional rhythm in the 80–90 bpm range, recorded off isoproterenol. No acute procedural complications were noted.

The patient remained asymptomatic for approximately 2 weeks after the procedure. During this period, a 7-day Holter monitor demonstrated an average heart rate of 116 bpm. Unfortunately, by the 3-month follow-up, she reported a recurrence of symptoms, including chest pain and shortness of breath, which closely resembled her preablation presentation. These symptoms persisted through the 6-month follow-up, indicating a return of her clinical syndrome despite initial postprocedural improvement.

## Discussion

In this case, focal PFA was used to modify the sinus node near the phrenic nerve safely, achieving acute procedural success without phrenic nerve injury; however, symptom recurrence was observed a few months after the procedure. Several factors contribute to the challenges and lower success rates of endocardial ablation of the sinus node. The sinoatrial node is a long, complex, and mainly epicardial structure extending from the superior vena cava to the high right atrium, with multiple potential sites of earliest activation.^10^ In addition, the close proximity of the right phrenic nerve to the sinoatrial node poses a risk of phrenic nerve damage during RF ablation or cryoablation. Phrenic nerve sparing techniques such as intrapericardial balloon can help minimize this risk.^11^ However, these methods are technically demanding, particularly in patients with previous cardiothoracic interventions. Previous reports have shown promising outcomes with combined endocardial and epicardial sinus node modification, which had already been attempted for this patient.^12^ Given these challenges, the need for safer, innovative procedural approaches is needed.

PFA achieves its effects by inducing irreversible electroporation of cardiac myocytes, effectively eliminating arrhythmogenic properties in cardiac tissue while preserving the function of nearby structures.^11^^,^^13^ This unique safety profile, particularly in regard to the phrenic nerve, makes PFA a promising option along with traditional thermal ablation methods. Currently, commercially available PFA systems in the United States are designed mainly for pulmonary vein isolation and lack the capability for focal ablation. However, investigational PFA catheters, such as the one used in this case with a relatively narrow tip and integrated with 3D electroanatomic mapping, allow for more targeted ablation and can deliver both PFA and RF energy. Contact force information ensured precise catheter stability and lesion delivery near the phrenic nerve. In addition, 3D electroanatomic mapping enhanced visualization and targeted ablation, improving procedural accuracy and safety. This adaptability makes focal PFA a suitable option for complex arrhythmias where phrenic nerve proximity is challenging.

In a recent case report, Kerley and colleagues^14^ (2024) demonstrated that sinus node modification using PFA can be performed safely and effectively; however, they used a broad pentaspline catheter (FARAPULSE) rather than focal PFA. Another case series evaluating PFA near the phrenic nerve found no evidence of long-term nerve damage.^15^

Although acute heart rate reductions were observed, the long-term efficacy was not achieved in this patient. Although this may suggest limited long-term efficacy, conclusions regarding procedural success or failure should be made cautiously, especially in light of the known challenges of durable sinus node modification via the endocardial approach. Several factors may have contributed to the lack of durable benefit, including insufficient lesion depth and incomplete transmurality in regions with thicker atrial tissue or epicardial origin. It is also important to consider that PFA lesions may include a zone of reversible electroporation surrounding the core area of irreversible injury.^16^ This transient functional impairment may explain the observed acute success with subsequent return of sinus activity. It is also possible that the acute response reflected modulation of local autonomic or vascular factors, such as sinus nodal artery vasoreactivity that has been reported with the use of PFA rather than true sinus node ablation.^17^

For future cases, we suggest adopting a more aggressive procedural approach to improve lesion durability, including optimized energy delivery parameters, broader lesion sets, and consideration of complementary strategies to improve the durability of sinus node modification procedures. Furthermore, refinement of procedural endpoints with respect to either PFA or RF, expanded isoproterenol stimulation protocols, and the adjunctive use of a nitroglycerin test to evaluate vasoreactivity may provide additional insights into the mechanism of acute suppression and help differentiate transient vascular effects from permanent nodal modification. Potentially, integration of lesion assessment tools and consideration of adjunctive epicardial approaches may further improve long-term outcomes. In addition, a better understanding of lesion depth, transmurality, and tissue response to PFA in this region is warranted.

## Conclusion

This case demonstrates the safety of focal PFA for sinus node modification in regions at a high risk of phrenic nerve injury. Although acute procedural success was achieved without complications, the recurrence of symptoms during follow-up highlights the need for further evaluation of long-term efficacy. Given its favorable safety profile in regard to phrenic nerve injury, focal PFA may offer a valuable alternative for managing focal arrhythmias in anatomically sensitive areas, potentially in patients with drug-refractory IST. Future studies should focus on optimizing lesion durability, refining procedural techniques, and exploring expanded strategies, including epicardial PFA, to enhance treatment outcomes in this complex patient population.

## Disclosures

The authors have no conflicts of interest to disclose.
